# Retrospective determination of whether famine existed in Niger, 2005: two stage cluster survey

**DOI:** 10.1136/bmj.a1622

**Published:** 2008-10-02

**Authors:** Avid Reza, Basia Tomczyk, Victor M Aguayo, Noel M Zagré, Kadadé Goumbi, Curtis Blanton, Leisel Talley

**Affiliations:** 1 International Emergency and Refugee Health Branch, Centers for Disease Control and Prevention, 4770 Buford Highway NE (MS F-60), Atlanta GA 30341, USA; 2 UNICEF, New Delhi, India; 3 UNICEF, Niamey, Niger; 4 Tuberculosis Programme, United Nations Development Programme, Niger

## Abstract

**Objective** To apply the famine scale by Howe and Devereux to the situation in Niger, west Africa, in 2005 to retrospectively determine whether famine existed.

**Design** Two stage cluster survey.

**Setting** Survey of households in each of Niger’s eight regions.

**Participants** 4003 households.

**Main outcome measures** Crude mortality, mortality in children under 5, and the proportion of caregivers both nationally and regionally adopting coping strategies to deal with insufficient food needs.

**Results** The estimated national crude mortality rate was 0.4 (0.4 to 0.5) deaths per 10 000 per day and under 5 mortality rate was 1.7 (1.4 to 1.9) deaths per 10 000 per day. Nationally, 22.3% (95% confidence interval 19.9% to 24.8%) of caregivers of under 5s did not resort to any coping strategies to deal with insufficient food needs. Reversible coping strategies were, however, used by 5.8% (4.7% to 7.0%) of caregivers, whereas 49.4% (46.9% to 51.8%) relied on irreversible coping strategies and 22.6% (20.0% to 25.4%) on survival strategies.

**Conclusion** On the basis of the famine scale proposed by Howe and Devereux, most regions in Niger experienced food crisis conditions and some areas approached famine proportions.

## Introduction

Food security in terms of availability and access became a widespread and pressing issue in 2008. This year has been marked by food riots in some of the poorest countries, sparked by hunger, and the prediction of an impending global food crisis.^1^ But how is a food crisis defined and what determines a famine? Controversy about the usage of the term famine has had unfortunate consequences for response and accountability in several recent humanitarian emergencies.^2^ The absence of universal benchmarks or criteria to identify famine conditions creates uncertainty about the magnitude of the crisis, resulting in delays and inappropriate responses.^2^ ^3^ In the context of the current global food crisis, with dramatic price increases and reduced accessibility to food in already food insecure populations,^1^ it may become even more critical to develop an internationally accepted definition of famine to guide humanitarian response and funding and to enforce accountability.

The famine scale proposed by Howe and Devereux offers a new approach to defining famine, based on scales of intensity and magnitude.^2^ ^3^ Intensity refers to the severity of the crisis in a given area at a specific time, and magnitude describes the aggregate impact of the entire crisis. The intensity level in a given population is based on a combination of anthropometric and mortality indicators and on descriptors of food security. These descriptors include coping strategies adopted by the affected population, and stability of the market and food prices. The intensity scale is used to assign population areas within a country to a level, from 0 (food secure conditions) to 5 (extreme famine conditions). Intensity levels of 3 (famine conditions) or above register as a famine on the magnitude scale, which ranges from minor famine to catastrophic famine. The magnitude scale is determined retrospectively by measuring excess mortality caused by the crisis.

We applied the Howe and Devereux famine scale to the humanitarian crisis in Niger, west Africa, in 2005 to determine if a famine occurred. Media reports of the famine conditions in Niger left little doubt of a humanitarian crisis but the scale and severity of the crisis and whether it reached famine proportions was disputed.

Along with a life expectancy of 45 years and a mortality rate in under 5s of 256 deaths per 1000 live births, Niger has some of the worst health indicators in the world.^4^ In 2005 it received the lowest rank (177th) on the human development index scale.^5^ The situation was exacerbated by massive poverty and recurring droughts. Of the approximately 11.7 million people living in Niger,^6^ the World Food Programme of the United Nations estimated that 2.5 million people in agropastoral areas experienced food insecurity in 2005 as a result of price increases and lack of access to food.^7^

International agencies raised concerns about the increasing admission rates of malnourished children in therapeutic feeding centres in Maradi region between February and April 2005.^8^ Local surveys in the Maradi and Tahoua regions between April and May 2005 showed that the prevalence of global acute malnutrition among children aged 6 to 59 months exceeded the 15% critical threshold established by the World Health Organization.^9^ ^10^ ^11^ It remained unclear whether these reports represented a localised event or if the crisis extended to regional and even national levels; the question of a current or imminent famine was also uncertain.^12^ Thus the scale and severity of the food crisis in Niger remained in dispute.

A proportionate and effective response to the crisis depended on an accurate determination of the extent of the crisis. Consequently from September to October 2005 the Centers for Disease Control and Prevention and Unicef, in collaboration with the Ministry of Health of Niger and non-governmental organisations, carried out a nationwide, population based nutrition survey. The objectives of this survey were to determine the prevalence of acute malnutrition and whether famine conditions were present and to identify potential underlying causes of malnutrition among children. Findings from this survey indicated that an acute nutrition crisis was taking place in the context of persistently high levels of acute and chronic malnutrition among young children.^13^ According to Howe and Devereux, however, the prevalence of global acute malnutrition alone is not sufficient to predict or to define famine.^2^

We applied the famine scale to the situation in Niger in 2005, by assessing mortality and household coping strategies with rates of malnutrition, to retrospectively determine whether famine existed.

## Methods

To obtain estimates at both regional and national level we carried out surveys in Niger’s eight regions: Agadez, Diffa, Dosso, Maradi, Tahoua, Tillaberi, Zinder, and the capital Niamey. We used a stratified two stage cluster survey design. The study population and sampling frame consisted of the entire population of Niger based on the 2001 census. Given the complexity of sampling the nomad population, estimated at 5% of the total population, we excluded that group from the sample.

Assuming a design effect of 1.7, we calculated that we would need a sample size of 470 children aged 6 to 59 months for each region to achieve a precision of 5% around an estimated 20%^10^ prevalence of global acute malnutrition, with 95% confidence intervals. To account for a 10% non-response rate we adjusted the final sample size to 520 children. Assuming that each household had one child aged 6 to 59 months, we determined that a total sample size of 520 households was required for each region; we therefore selected 26 clusters of 20 households for each region. We estimated that the combined sample from the eight regions would yield a total sample of 4160 households, resulting in a precision of 2% around an estimated national prevalence of global acute malnutrition of 20%, with a 95% confidence interval. In addition, this sample size resulted in a precision of 0.4 deaths per 10 000 per day, with a 95% confidence interval around the estimated crude mortality and under 5 mortality rates.

In the first sampling stage we selected 26 village level clusters in each region where the probability of selection was proportionate to the population size. In the second stage we used systematic random sampling to select 20 households per cluster. A household was defined as a group of people who lived together in the same housing unit, who ate food prepared in the same cooking pot, and who recognised the same person as the head of the household.

The team obtained oral consent for participation from the head of each selected household. To estimate mortality we included all selected households in the survey, regardless of whether a child aged 6 to 59 months was a household member. If the head of the household or another adult was not present at the first visit, researchers visited again. If after two visits no adult was available, the household was omitted and not replaced. A questionnaire at household level was administered to the head of the household, which included a census of the household. The remainder of the questionnaire was administered to mothers or caregivers of children under 5 living in the household. Caregivers were asked about the coping strategies used for dealing with insufficient food needs for the household since the beginning of the last “hungry season” in 2005. Additionally, we collected anthropometric measurements on children aged 6 to 59 months. Children who were admitted to therapeutic feeding centres were weighed and measured at the centre.

National staff were trained as interviewers and as anthropometrists. Data were recorded in French, Djerma, or Hausa (the three main languages in Niger) on a questionnaire that had been translated and back translated in each of the languages. We based the questionnaire on instruments used in previous emergencies as well as on standardised national instruments used in Niger.^14^ To reflect culturally specific attitudes and practices in Niger we further modified these questions on the basis of interviews with key informants and findings from the pilot.

To assist in determining the age of children and the dates of any deaths of household members, interviewers used a local calendar of events well known to Niger residents. A household census was taken to determine the total number of people and the number of under 5s present since 21 January 2005—the day of Tabaski, a major religious event in the country. We recorded deaths in the household between 21 January 2005 and the date of the interview, and the month of death. We used a series of structured questions to assign cause of death among under 5s into one of eight categories based on a standard verbal autopsy.^15^ In addition to determining cause of death, we asked questions on the child’s appearance at the time of death to determine if malnutrition may have been a contributing factor.

Questions on the various coping strategies of the households were based on previously documented strategies.^16^ To reflect culturally specific coping practices in Niger we then further modified these questions on the basis of interviews with key informants. Caregivers of under 5s were directly asked about coping strategies.

We stratified the coping strategies for dealing with insufficient food needs since the beginning of the last hungry season in 2005 into four mutually exclusive categories: no coping strategies, reversible adaptive strategies that should not harm future livelihoods, irreversible coping strategies that threatened future livelihoods, and survival strategies involving disengagement from any normal livelihood systems and focusing purely on survival (see box).

**Coping strategies used by households in Niger**

**Reversible adaptive strategies**

Changing consumption levels—for example, reducing frequency of meals
Changing diet—for example, collecting wild foods
Selling small livestock
Selling unproductive assets (personal items not including jewellery, which is seen as an investment and therefore usually sold last)
Seasonal migration in search of employment or food
Seeking employment for food only
Borrowing food or money from family or friends
Pawning assets for food or money
Begging (once, sometimes)

**Irreversible coping strategies**

Selling large livestock
Selling young female livestock
Selling production tools
Selling jewellery
Purchasing food on credit
Begging (often)

**Survival strategies**

Severely reducing consumption levels
Selling land
Unusual migration of whole household

The severity of the individual coping strategies was based on interviews with key informants. We analysed the caregivers’ responses and assigned each caregiver to one of the four categories for coping strategies. The most severe strategy adopted by caregivers determined their category placement. We estimated the proportion of caregivers both regionally and nationally adopting each coping strategy.

Data were entered using Epi Info software version 6.04b and managed using SAS version 9.1. To account for weighting of the variables and the complex sample design (stratification and clustering at the village level) we used SAS callable SUDAAN version 9.01. We calculated sampling weights by multiplying the inverse probability for selection of a person within a household multiplied by a post-stratification adjustment on the basis of the census population of the region. The procedure Proc Ratio was used to compute 95% confidence intervals for mortality. We based the number of recall days for each person on the date of the interview minus the start of the recall period (21 January 2005). For live births and deaths during the recall period we estimated the number of recall days as half of the recall period. For proportions we computed confidence intervals using Proc Crosstab. To test the difference in coping strategies between urban and rural regions we ran a *t* test using Proc Descript.

## Results

From 17 September to 14 October 2005 information was collected by survey from 4003 of 4160 households (overall response rate 95.6%) in eight regions of Niger. Information on health was gathered on 5309 children aged under 5. The mean size of households nationally was 6.9, and under 5s constituted 21.0% of household members.

### Mortality

The estimated crude mortality rate nationally was 0.4 deaths per 10 000 per day (95% confidence interval 0.4 to 0.5) whereas regionally it ranged from 0.2 deaths per 10 000 per day (0.1 to 0.3) in Agadez to 0.7 deaths per 10 000 per day (0.5 to 0.9) in Zinder (table 1). The under 5 mortality rate nationally was 1.7 (1.4 to 1.9) deaths per 10 000 per day whereas regionally it ranged from 0.3 (0.1 to 0.7) deaths per 10 000 per day in Agadez to 2.2 (1.4 to 3.0) deaths per 10 000 per day in Zinder (table 1).

**Table 1 tbl1:** Crude mortality and under 5 mortality rates by region in Niger, 17 September to 14 October 2005

Region	Crude mortality			Under 5 mortality	
	No in sample	Deaths/10 000/day (95% CI)*		No in sample	Deaths/10 000/day (95% CI)*
Agadez	3213	0.2 (0.1 to 0.3)		642	0.3 (0.1 to 0.7)
Diffa	2654	0.4 (0.1 to 0.6)		565	1.4 (0.2 to 2.6)
Dosso	4048	0.3 (0.2 to 0.5)		827	1.2 (0.7 to 1.8)
Maradi	3722	0.3 (0.2 to 0.5)		884	1.3 (0.8 to 1.8)
Tahoua	3219	0.5 (0.3 to 0.6)		732	2.1 (1.4 to 2.9)
Tillaberi	3932	0.5 (0.3 to 0.6)		850	1.7 (1.1 to 2.3)
Zinder	3118	0.7 (0.5 to 0.9)		720	2.2 (1.4 to 3.0)
Niamey	3409	0.4 (0.2 to 0.6)		518	1.1 (0.4 to 1.8)
National	27 315	0.4 (0.4 to 0.5)		5738	1.7 (1.4 to 1.9)

*Weighted results.

The cause specific mortality among under 5s showed that most deaths nationally from known causes were reported as diarrhoea (30.2%, 95% confidence interval 22.7% to 38.8%), acute respiratory illness (20.1%, 13.6% to 28.6%), and meningitis (14.1%, 9.7% to 20.1%). Insufficient sample size prevented the determination of regional variations. Of the children who died during the recall period, 51.6% (42.2% to 61.0%) were perceived by their caregiver as malnourished.

### Coping strategies

Since the beginning of the last hungry season in Niger in 2005, coping strategies were not used by 22.3% (19.9% to 24.8%) of caregivers nationally (table 2). Also nationally 5.8% (4.7% to 7.0%) of caregivers of under 5s relied on reversible coping strategies, whereas 49.4% (46.9% to 51.8%) relied on irreversible coping strategies and 22.6% (20.0% to 25.4%) on survival strategies. A similar pattern was observed in the seven predominantly rural regions (except Niamey), in which most caregivers relied on irreversible coping strategies. Among these seven regions the proportion of caregivers relying on irreversible strategies varied from 35.4% (28.3% to 43.1%) in Agadez to 64.7% (58.7% to 70.3%) in Zinder, and the proportion relying on survival strategies ranged from 14.2% (9.7% to 20.4%) in Diffa to 33.0% (24.4% to 43.0%) in Maradi. In contrast with the predominantly rural regions, 56.2% (49.8% to 62.4%) of caregivers in Niamey, a largely urban population, did not have to resort to any coping strategies. Compared with each of the other seven regions, the proportion of caregivers in Niamey not using any coping strategies differed statistically (P<0.001 for all seven *t* tests).

**Table 2 tbl2:** Proportion of caregivers adopting varying degrees of coping strategies by region in Niger, 17 September to 14 October 2005

Region	Degree (%) of coping strategies (95% CI)*				
	No in sample	None	Reversible	Irreversible	Survival
Agadez	367	35.2 (27.7 to 43.5)	7.2 (4.5 to 11.4)	35.4 (28.3 to 43.1)	22.3 (16.1 to 29.9)
Diffa	328	32.2 (22.7 to 43.4)	3.5 (1.6 to 7.8)	50.1 (41.1 to 59.1)	14.2 (9.7 to 20.4)
Dosso	488	30.2 (23.6 to 37.7)	7.1 (4.6 to 11.0)	43.4 (36.8 to 50.2)	19.3 (14.6 to 25.0)
Maradi	521	18.7 (12.6 to 27.0)	4.4 (2.5 to 7.6)	43.8 (37.2 to 50.7)	33.0 (24.4 to 43.0)
Tahoua	427	26.9 (21.5 to 33.0)	5.5 (3.3 to 9.0)	46.2 (42.2 to 50.3)	21.5 (16.0 to 28.2)
Tillaberi	483	18.2 (14.1 to 23.3)	5.1 (2.4 to 10.8)	55.7 (50.0 to 61.2)	21.0 (17.7 to 24.6)
Zinder	414	8.2 (5.1 to 12.8)	6.0 (4.4 to 8.2)	64.7 (58.7 to 70.3)	21.1 (16.5 to 26.6)
Niamey	350	56.2 (49.8 to 62.4)	11.3 (8.7 to 14.5)	26.4 (22.4 to 30.8)	6.1 (4.2 to 8.8)
Total	3378	22.3 (19.9 to 24.8)	5.8 (4.7 to 7.0)	49.4 (46.9 to 51.8)	22.6 (20.0 to 25.4)

*Weighted results.

## Discussion

Using the famine scale by Howe and Devereux we retrospectively determined that a food crisis was under way at the time of the household survey in Niger, 2005. This crisis affected children in each region to varying degrees.^13^ In four of the eight regions the prevalence of global acute malnutrition among children aged 6 to 59 months exceeded the critical threshold of 15% established by WHO,^11^ indicating the need for an immediate humanitarian response to prevent further increases in malnutrition, morbidity, and mortality in children.^13^ Yet these results alone did not answer whether a famine had existed.

By applying the famine intensity scale and by examining the anthropometric findings in conjunction with indicators of mortality and descriptors of food security we can better determine whether the crisis reached famine proportions.^2^ Despite the critical levels of acute malnutrition reported in Diffa, Maradi, Tahoua, and Zinder,^13^ the prevalence of global acute malnutrition in these regions at the time of the survey was below the 20% threshold (level 3, or famine conditions).^2^ The levels of global acute malnutrition in these four regions were consistent with food crisis conditions (level 2). Furthermore, crude mortality rates both nationally and regionally remained below the benchmark of 1 death per 10 000 per day, as required for famine conditions.^2^ The crude mortality rates in Tahoua, Tillaberi, and Zinder were between 0.5 and 1 death per 10 000 per day—consistent with food crisis conditions, whereas the remaining regions experienced crude mortality rates consistent with conditions for food insecurity (level 1). Although crude mortality rates did not indicate famine conditions, the survey results suggest that malnutrition did play an important part in child mortality, given that 51.6% (95% confidence interval 42.2% to 61.0%) of under 5s who died during the recall period were reported by their caregivers as malnourished. In comparison, 15.6% of the global deaths among under 5s in 1994 were attributed to malnutrition.^17^ During famine, malnutrition plays a much larger part in child mortality. For example, during the famine in Ethiopia (1999-2000) malnutrition contributed to more than 70% of deaths among under 5s.^18^ Taken together, the anthropometry and mortality indicators suggest that Diffa, Maradi, Tahoua, Tillaberi, and Zinder experienced food crisis conditions, whereas the remaining three regions experienced food insecurity conditions.

The famine intensity scale also takes into consideration the level of food security descriptors.^2^ At the onset of the crisis, food insecurity was undoubtedly a problem; disagreement remained, however, about the magnitude of the crisis and the effect on food availability as a result of drought, locust invasion, and high prices.^12^ ^19^ The food security assessment carried out jointly by the government of Niger, the Food and Agricultural Organisation, the World Food Program, and the Famine Early Warning Systems Network in April 2005 indicated that about 67% of the people living in agropastoral areas were highly vulnerable to food insecurity.^19^ Coverage by the media and reports from non-governmental organisations claimed famine conditions, but the joint food security assessment concluded that the crisis did not reach famine proportions.^19^

The survey results show that during the crisis more than 50% of caregivers of under 5s in all regions except Niamey relied on irreversible or survival coping strategies. In all regions except Niamey irreversible coping strategies were the most common means to deal with the crisis. According to the famine intensity scale, the adoption of irreversible coping strategies is most consistent with a food crisis (level 2).^2^ In Agadez, Maradi, Tahoua, Tillaberi, and Zinder, however, more than 20% of caregivers of under 5s resorted to survival strategies. Although the famine intensity scale does not specify what percentage of the affected population has to rely on survival strategies to constitute famine conditions, the large proportion of caregivers using survival strategies seems to indicate that in some regions famine may have been imminent.

During the crisis many raised concerns that Unicef, non-governmental organisations, and the international media were exaggerating the magnitude of the situation.^19^ Our results indicate that the crisis was not just a localised event but was widespread. Most regions in Niger experienced food crisis conditions, and the adoption of survival strategies by caregivers further suggests that many regions were potentially on the threshold of a famine. Moreover, the large scale humanitarian response may well have prevented the crisis from reaching famine proportions. Before 2005 few feeding programmes were available in Niger. In June 2005, Médecins Sans Frontières operated one therapeutic feeding programme. By the time we did our survey, the humanitarian response was well under way; by December 2005 about 253 therapeutic feeding programmes provided care to severely malnourished children and 583 supplementary feeding programmes provided care for those who were moderately malnourished. These interventions may have prevented excess morbidity and mortality and may have explained why, despite the adoption of survival strategies typical of famine conditions, the anthropometry and mortality results suggested a food crisis and not a famine.

### Limitations

Our study has several limitations. Firstly, an estimate of retrospective mortality is subject to recall bias. We attempted to minimise this bias by limiting the recall period to eight months and by defining the beginning of the period using Tabaski, a well known religious holiday. Secondly, in the absence of a reliable death registry system or functioning surveillance system, we relied on oral reports of interviewed household members to determine cause of death in under 5s. Although we used a series of structured questions based on the WHO criteria for verbal autopsy to improve the accuracy of the data and to help classify cause of death, some misclassification may have occurred. Thirdly, questions on coping strategies were administered to caregivers of under 5s, therefore these results are not representative of all households nationally. Finally, in the absence of an agreed and standardised analysis tool for assessing household coping strategies, we consolidated these strategies into mutually exclusive categories and assigned each respondent to one of them. This approach does not, however, capture the complex range of strategies to which each household resorted. The coping strategies index has been suggested to better take into account the severity and frequency of each of the coping strategies used in a household and results in a simple numerical score.^20^ Without a baseline score, however, the numerical value by itself cannot be interpreted and therefore it was not helpful in assessing the severity of the crisis on the basis of the coping strategies in this paper.

### Conclusion

On the basis of the famine scale, most regions in Niger in 2005 experienced food crisis conditions and some areas approached famine proportions. The absence of a universally accepted definition of famine contributed to disagreements about the magnitude and severity of the problem. In the past, disagreements over the terminology have contributed to delayed interventions and inequitable distribution of resources.^2^ This highlights the need for an internationally agreed definition of famine and for clear guidelines on the timing and magnitude of a response. Although the utility of the famine scale by Howe and Devereux needs rigorous assessment, it does provide more objective criteria than previous approaches. It also moves from a binary concept of “famine” or “no famine” to a graduated approach that captures a range of food insecurity situations.^2^ One potential use of the famine scale is in conjunction with early warning systems, to help guide the level of humanitarian response and to provide a greater opportunity to recognise a potential famine.^3^ Nutrition surveys such as ours, with data collected on malnutrition and mortality as well as on coping strategies, improve the ability to triangulate different types of information to determine the true magnitude of a crisis. A determination of the scale and severity of a crisis and whether it reaches famine proportions is important at the time of the actual crisis for ensuring a proportionate and timely response. Additionally, it is essential that the context of the situation be concurrently assessed to assist in the situational analysis. This might include local variations that may influence morbidity and mortality as well as how communities and households cope both positively and detrimentally with shocks. It is equally important to examine the basic and underlying causes of malnutrition, which do not necessarily arise solely from insufficient access to food or problems with food production. The causes of malnutrition in children are complex and multifaceted, including but not limited to suboptimal feeding practices; inadequate access to essential health services; women’s lack of access to education, information, and decision making; and poverty.^21^ ^22^ ^23^ ^24^ ^25^ Examination of multiple aspects of a crisis can strengthen and ensure an appropriate response. This type of analysis and assessment may become even more important as the global community faces the challenge of increasing food shortages in the most vulnerable populations.

**What is already known on this topic**

A lack of universal criteria to identify famine creates uncertainty about the size of the crisis, resulting in delays and inappropriate responses
The scale and severity of the food crisis in Niger in 2005 and whether it reached famine proportions was disputed

**What this study adds**

On the basis of the famine scale by Howe and Devereux, most regions in Niger in 2005 experienced food crisis conditions, and some areas approached famine
The scale provides more objective criteria than do previous approaches and along with early warning systems may help guide the level of response

## Notes

We thank the field team members who participated in carrying out this national survey; Alichina Kourgueni for his expertise and logistical support; Sybille Gumucio and Robert Ndamobissi for coordinating the survey operations; Jonathan Seikmann for data management; Abba Kakk, Issoufou Lamissi, Bintou Tidjane, Théophile Bansimba, Baro Mohammed, and Hamadou Boureima for supervising and coordinating the field teams; Ibrahim Combasse and Alio Maidaji for translation; Yacouba Mamdou for providing oversight for data entry; Aminatou Alzouma, Foureratou Ekade, Makinta Lalla, Rachidou Katchi Amadou, Ramatou Alou Mamane, and Naroua Mavaki for data entry; and Mark Anderson for his critical input of the manuscript. KG was affiliated with the Ministry of Health, Niger during the survey. The opinions presented in this paper are those of the authors and do not necessarily reflect those of the institutions with which they are affiliated.

Ethical approval: Not required as the primary intent of the survey was determined to be non-research.

Provenance and peer review: Not commissioned; externally peer reviewed.

## Notes

**Cite this as: ***BMJ* 2008;337:a1622
